# Benzene-1,3,5-tricarb­oxy­lic acid–1,10-bis­(1,2,4-triazol-1-yl)deca­ne–water (1/1/2)

**DOI:** 10.1107/S1600536810044673

**Published:** 2010-11-06

**Authors:** Lian-Peng Zhao

**Affiliations:** a BoHai University, JinZhou, LiaoNing 121013, People’s Republic of China

## Abstract

In the title 1:1:2 association, C_14_H_24_N_6_·C_9_H_6_O_6_·2H_2_O, the alkyl chain in the 1,10-bis­(1,2,4-triazol-1-yl)decane mol­ecule adopts an extended conformation and the dihedral angle between the aromatic rings is 10.28 (13)°. The benzene-1,3,5-tricarb­oxy­lic acid mol­ecule is close to being planar (r.m.s. deviation = 0.052 Å). In the crystal, the components are linked by O—H⋯O and O—H⋯N hydrogen bonds, generating a layered network.

## Related literature

For backgound to supra­molecular networks, see: Ma & Coppens (2003 ▶).
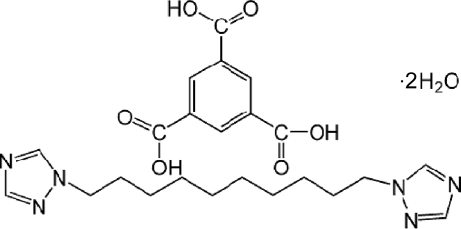


## Experimental

### 

#### Crystal data

C_14_H_24_N_6_·C_9_H_6_O_6_·2H_2_O
*M* *_r_* = 522.56
Triclinic, 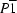

*a* = 10.7715 (6) Å
*b* = 11.4405 (6) Å
*c* = 11.7458 (6) Å
α = 101.790 (4)°
β = 105.800 (4)°
γ = 92.740 (4)°
*V* = 1355.13 (12) Å^3^
*Z* = 2
Mo *K*α radiation
μ = 0.10 mm^−1^
*T* = 293 K
0.32 × 0.29 × 0.2 mm

#### Data collection

Oxford Diffraction Gemini R Ultra CCD diffractometer
Absorption correction: multi-scan (*CrysAlis CCD*; Oxford Diffraction, 2006 ▶) *T*_min_ = 0.952, *T*_max_ = 0.984
8774 measured reflections
4921 independent reflections
1989 reflections with *I* > 2σ(*I*)
*R*_int_ = 0.030

#### Refinement

*R*[*F* ^2^ > 2σ(*F* ^2^)] = 0.036
*wR*(*F* ^2^) = 0.052
*S* = 0.88
4921 reflections
355 parameters
9 restraints
H atoms treated by a mixture of independent and constrained refinement
Δρ_max_ = 0.13 e Å^−3^
Δρ_min_ = −0.12 e Å^−3^

### 

Data collection: *CrysAlis CCD* (Oxford Diffraction, 2006 ▶); cell refinement: *CrysAlis CCD*; data reduction: *CrysAlis RED* (Oxford Diffraction, 2006 ▶); program(s) used to solve structure: *SHELXS97* (Sheldrick, 2008 ▶); program(s) used to refine structure: *SHELXL97* (Sheldrick, 2008 ▶); molecular graphics: *SHELXTL* (Sheldrick, 2008 ▶); software used to prepare material for publication: *SHELXL97*.

## Supplementary Material

Crystal structure: contains datablocks I, global. DOI: 10.1107/S1600536810044673/hb5722sup1.cif

Structure factors: contains datablocks I. DOI: 10.1107/S1600536810044673/hb5722Isup2.hkl

Additional supplementary materials:  crystallographic information; 3D view; checkCIF report

## Figures and Tables

**Table 1:** Hydrogen-bond geometry (Å, °)

*D*—H⋯*A*	*D*—H	H⋯*A*	*D*⋯*A*	*D*—H⋯*A*
O2—H2*WA*⋯O1*W*	0.89 (2)	1.66 (2)	2.549 (2)	177 (3)
O4—H4*WA*⋯O2*W*	0.88 (2)	1.70 (2)	2.571 (2)	169 (3)
O5—H5*WA*⋯N6^i^	0.99 (2)	1.59 (2)	2.5750 (19)	174 (3)
O1*W*—H1*AW*⋯O3^ii^	0.87 (2)	1.86 (2)	2.711 (2)	163 (3)
O1*W*—H1*BW*⋯O1^iii^	0.78 (2)	1.99 (2)	2.757 (2)	170 (3)
O2*W*—H2*BW*⋯N3^ii^	0.90 (2)	2.12 (2)	2.875 (2)	140 (3)
O2*W*—H2*AW*⋯O6^iv^	0.80 (2)	2.05 (2)	2.841 (2)	170 (3)

Symmetry codes: (i) 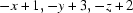; (ii) 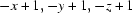; (iii) 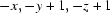; (iv) 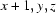.

**Fig. 1.**
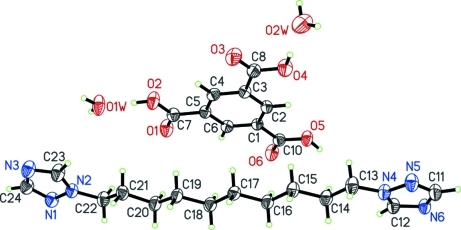
The molecular structure of (I) with displacement ellipsoids drawn at the 30% probability level.

**Fig. 2.**
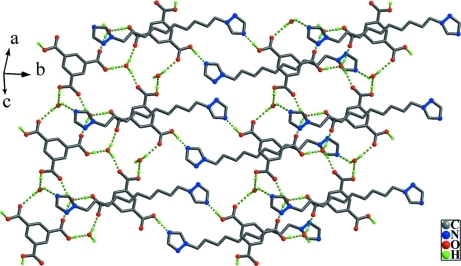
C—H···O interactions (dotted lines) in the crystal of the title compound.

## supplementary crystallographic information

### Comment

There are numerous framework building blocks from benzene-1,3,5-tricarboxylic acid in the synthesis of organic supramlecular solids because of its rigidity and triangular geometry (Ma & Coppens, 2003). Up to now, the bis(1,2,4-triazol-1-yl)decane ligand, as a flexible ligand, is rarely investigated in constructing supramlecular compounds.

In the crystal structure of the title compound, (I), there are one benzene-1,3,5-tricarboxylic acid, one bis(1,2,4-triazol-1-yl)decane and two water molecules (Fig. 1). O1W water molelcule acts as both acceptor and donor, forming three hydrogen bonds, which link adjacent benzene-1,3,5-tricarboxylic acid molecules. As well as O2W water molecule, which also acts as both acceptor and donor, linking two adjacent benzene-1,3,5-tricarboxylic acid and one adjacent bis(1,2,4-triazol-1-yl)decane molecules. Thus, benzene-1,3,5-tricarboxylic acid and bis(1,2,4-triazol-1-yl)decane molecules are linked through strong intermolecular O—H···O and O—H···N interactions, forming a two dimensional supramolecular layer (Fig. 2).

### Experimental

Benzene-1,3,5-tricarboxylic acid (0.042 g, 0.2 mmol) and bis(1,2,4-triazol-1-yl)decane (0.055 g, 0.2 mmol) was added in a beaker of a methanol (10 ml) and water (5 ml) solution. The mixture was heated to 60 °C and held at that temperature for 10 minutes, then cooled to room temperature and filtered. The filtrate was left in a beaker for two days and colourless blocks of (I) were isolated.

### Refinement

C–bound H–atoms were geometrically positioned (C—H 0.93 Å) and refined using a riding model, with *U*_iso_ = 1.2*U*_eq_ (C). The water H atoms were located in a difference Fourier map and refined with *U*_iso_(H)= 1.5Ueq(O).

### Crystal data

**Table:**

C_14_H_24_N_6_·C_9_H_6_O_6_·2H_2_O	*Z* = 2
*M**_r_* = 522.56	*F*(000) = 556
Triclinic, *P*1	*D*_x_ = 1.281 Mg m^−^^3^
Hall symbol: -P 1	Mo *K*α radiation, λ = 0.71073 Å
*a* = 10.7715 (6) Å	Cell parameters from 4921 reflections
*b* = 11.4405 (6) Å	θ = 3.0–25.4°
*c* = 11.7458 (6) Å	µ = 0.10 mm^−^^1^
α = 101.790 (4)°	*T* = 293 K
β = 105.800 (4)°	Block, colorless
γ = 92.740 (4)°	0.32 × 0.29 × 0.2 mm
*V* = 1355.13 (12) Å^3^	

### Data collection

**Table:**

Oxford Diffraction Gemini R Ultra CCD diffractometer	4921 independent reflections
Radiation source: fine-focus sealed tube	1989 reflections with *I* > 2σ(*I*)
graphite	*R*_int_ = 0.030
Detector resolution: 10.0 pixels mm^-1^	θ_max_ = 25.4°, θ_min_ = 3.1°
ω scan	*h* = −12→11
Absorption correction: multi-scan (*CrysAlis CCD*; Oxford Diffraction, 2006)	*k* = −10→13
*T*_min_ = 0.952, *T*_max_ = 0.984	*l* = −14→11
8774 measured reflections	

### Refinement

**Table:**

Refinement on *F*^2^	Primary atom site location: structure-invariant direct methods
Least-squares matrix: full	Secondary atom site location: difference Fourier map
*R*[*F*^2^ > 2σ(*F*^2^)] = 0.036	Hydrogen site location: inferred from neighbouring sites
*wR*(*F*^2^) = 0.052	H atoms treated by a mixture of independent and constrained refinement
*S* = 0.88	*w* = 1/[σ^2^(*F*_o_^2^) + (0.0072*P*)^2^] where *P* = (*F*_o_^2^ + 2*F*_c_^2^)/3
4921 reflections	(Δ/σ)_max_ = 0.001
355 parameters	Δρ_max_ = 0.13 e Å^−^^3^
9 restraints	Δρ_min_ = −0.12 e Å^−^^3^

### Special details

**Table:**

Geometry. All s.u.'s (except the s.u. in the dihedral angle between two l.s. planes) are estimated using the full covariance matrix. The cell s.u.'s are taken into account individually in the estimation of s.u.'s in distances, angles and torsion angles; correlations between s.u.'s in cell parameters are only used when they are defined by crystal symmetry. An approximate (isotropic) treatment of cell s.u.'s is used for estimating s.u.'s involving l.s. planes.
Refinement. Refinement of *F*^2^ against ALL reflections. The weighted *R*-factor *wR* and goodness of fit *S* are based on *F*^2^, conventional *R*-factors *R* are based on *F*, with *F* set to zero for negative *F*^2^. The threshold expression of *F*^2^ > 2σ(*F*^2^) is used only for calculating *R*-factors(gt) *etc*. and is not relevant to the choice of reflections for refinement. *R*-factors based on *F*^2^ are statistically about twice as large as those based on *F*, and *R*- factors based on ALL data will be even larger.

### Fractional atomic coordinates and isotropic or equivalent isotropic displacement parameters (Å^2^)

**Table:**

	*x*	*y*	*z*	*U*_iso_*/*U*_eq_	
C1	0.39170 (19)	0.95737 (17)	0.80707 (17)	0.0449 (5)	
C2	0.52268 (18)	0.95284 (16)	0.81530 (16)	0.0493 (6)	
H2	0.5829	1.0174	0.8625	0.059*	
C3	0.56376 (19)	0.85253 (17)	0.75336 (18)	0.0470 (5)	
C4	0.47358 (19)	0.75609 (17)	0.68424 (18)	0.0520 (6)	
H4	0.5010	0.6887	0.6428	0.062*	
C5	0.34384 (19)	0.75882 (17)	0.67614 (17)	0.0480 (6)	
C6	0.30284 (18)	0.86009 (17)	0.73798 (17)	0.0502 (6)	
H6	0.2153	0.8623	0.7328	0.060*	
C7	0.2450 (2)	0.65651 (19)	0.6038 (2)	0.0570 (6)	
C8	0.7031 (2)	0.8428 (2)	0.7614 (2)	0.0571 (6)	
C10	0.3452 (2)	1.06481 (18)	0.87083 (19)	0.0542 (6)	
C11	0.7731 (2)	1.65574 (19)	0.9521 (2)	0.0672 (7)	
H11	0.8421	1.7142	0.9951	0.081*	
C12	0.58551 (19)	1.56741 (18)	0.88510 (19)	0.0610 (6)	
H12	0.4967	1.5473	0.8688	0.073*	
C13	0.63731 (19)	1.38270 (17)	0.7527 (2)	0.0719 (7)	
H13A	0.6884	1.3262	0.7909	0.086*	
H13B	0.6645	1.3900	0.6820	0.086*	
C14	0.49697 (19)	1.33401 (16)	0.71237 (19)	0.0626 (6)	
H14A	0.4457	1.3881	0.6703	0.075*	
H14B	0.4682	1.3292	0.7829	0.075*	
C15	0.47559 (18)	1.20989 (16)	0.62827 (18)	0.0619 (6)	
H15A	0.5070	1.2150	0.5593	0.074*	
H15B	0.5262	1.1561	0.6713	0.074*	
C16	0.33408 (18)	1.15749 (16)	0.58242 (18)	0.0585 (6)	
H16A	0.2838	1.2103	0.5377	0.070*	
H16B	0.3021	1.1541	0.6514	0.070*	
C17	0.31336 (17)	1.03190 (15)	0.50045 (18)	0.0577 (6)	
H17A	0.3438	1.0356	0.4307	0.069*	
H17B	0.3650	0.9795	0.5446	0.069*	
C18	0.17162 (18)	0.97804 (16)	0.45616 (18)	0.0639 (6)	
H18A	0.1199	1.0303	0.4117	0.077*	
H18B	0.1410	0.9744	0.5259	0.077*	
C19	0.15155 (18)	0.85218 (15)	0.37448 (19)	0.0625 (6)	
H19A	0.1721	0.8572	0.3001	0.075*	
H19B	0.2111	0.8025	0.4152	0.075*	
C20	0.01312 (18)	0.79255 (16)	0.34285 (19)	0.0675 (7)	
H20A	−0.0460	0.8390	0.2969	0.081*	
H20B	−0.0097	0.7925	0.4172	0.081*	
C21	−0.00327 (18)	0.66361 (16)	0.26872 (19)	0.0631 (6)	
H21A	0.0140	0.6637	0.1919	0.076*	
H21B	0.0589	0.6179	0.3124	0.076*	
C22	−0.13937 (18)	0.60470 (17)	0.24526 (19)	0.0650 (6)	
H22A	−0.1547	0.6015	0.3223	0.078*	
H22B	−0.2013	0.6534	0.2059	0.078*	
C23	−0.0785 (2)	0.4098 (2)	0.1410 (2)	0.0772 (8)	
H23	0.0110	0.4297	0.1643	0.093*	
C24	−0.2628 (2)	0.3200 (2)	0.0669 (2)	0.0728 (7)	
H24	−0.3302	0.2597	0.0247	0.087*	
N1	−0.28416 (16)	0.42595 (17)	0.12176 (17)	0.0691 (6)	
N2	−0.16118 (17)	0.48321 (14)	0.16904 (15)	0.0574 (5)	
N3	−0.13877 (18)	0.30359 (15)	0.07500 (18)	0.0791 (6)	
N4	0.66373 (17)	1.49947 (14)	0.83772 (15)	0.0575 (5)	
N5	0.78695 (16)	1.55567 (16)	0.88053 (17)	0.0691 (6)	
N6	0.65112 (17)	1.66695 (14)	0.95827 (15)	0.0599 (5)	
O1	0.13154 (14)	0.65517 (12)	0.59815 (14)	0.0793 (5)	
O2	0.29321 (14)	0.56664 (13)	0.54589 (15)	0.0802 (5)	
O3	0.73875 (13)	0.75585 (13)	0.70565 (14)	0.0801 (5)	
O4	0.78183 (13)	0.93484 (14)	0.83381 (16)	0.0771 (5)	
O5	0.43572 (13)	1.15486 (12)	0.92566 (13)	0.0646 (4)	
O6	0.23315 (14)	1.06863 (11)	0.87170 (14)	0.0758 (5)	
O1W	0.12775 (14)	0.38874 (15)	0.41955 (18)	0.0959 (6)	
O2W	1.02243 (15)	0.90437 (15)	0.8626 (2)	0.1207 (8)	
H2WA	0.234 (2)	0.506 (2)	0.502 (3)	0.181*	
H4WA	0.863 (2)	0.925 (2)	0.834 (3)	0.181*	
H5WA	0.398 (3)	1.223 (2)	0.966 (2)	0.181*	
H1AW	0.156 (3)	0.333 (2)	0.372 (3)	0.181*	
H1BW	0.055 (2)	0.368 (2)	0.410 (3)	0.181*	
H2BW	1.046 (3)	0.855 (2)	0.914 (3)	0.181*	
H2AW	1.075 (3)	0.956 (2)	0.863 (3)	0.181*	

### Atomic displacement parameters (Å^2^)

**Table:**

	*U*^11^	*U*^22^	*U*^33^	*U*^12^	*U*^13^	*U*^23^
C1	0.0427 (13)	0.0458 (13)	0.0470 (15)	0.0068 (11)	0.0156 (11)	0.0084 (11)
C2	0.0460 (14)	0.0494 (14)	0.0501 (15)	0.0031 (11)	0.0144 (11)	0.0056 (11)
C3	0.0434 (13)	0.0477 (14)	0.0523 (16)	0.0036 (11)	0.0188 (12)	0.0105 (11)
C4	0.0589 (15)	0.0506 (14)	0.0528 (15)	0.0144 (12)	0.0246 (12)	0.0126 (12)
C5	0.0432 (13)	0.0477 (14)	0.0529 (16)	0.0007 (11)	0.0168 (12)	0.0079 (11)
C6	0.0442 (13)	0.0530 (14)	0.0566 (15)	0.0087 (12)	0.0211 (12)	0.0101 (12)
C7	0.0574 (15)	0.0597 (16)	0.0553 (16)	0.0080 (13)	0.0227 (14)	0.0072 (12)
C8	0.0507 (15)	0.0543 (16)	0.0676 (18)	0.0066 (13)	0.0199 (14)	0.0126 (13)
C10	0.0519 (15)	0.0519 (15)	0.0586 (16)	0.0098 (13)	0.0181 (13)	0.0086 (12)
C11	0.0554 (16)	0.0580 (16)	0.0782 (19)	−0.0045 (12)	0.0161 (13)	−0.0004 (13)
C12	0.0492 (14)	0.0534 (15)	0.0747 (18)	0.0044 (13)	0.0211 (13)	−0.0027 (13)
C13	0.0639 (16)	0.0571 (15)	0.0881 (19)	0.0031 (12)	0.0339 (14)	−0.0140 (13)
C14	0.0600 (14)	0.0530 (14)	0.0712 (17)	0.0043 (11)	0.0241 (13)	−0.0002 (12)
C15	0.0668 (15)	0.0513 (14)	0.0644 (16)	0.0018 (12)	0.0249 (13)	−0.0011 (12)
C16	0.0605 (14)	0.0504 (14)	0.0605 (16)	0.0053 (11)	0.0173 (12)	0.0039 (12)
C17	0.0586 (14)	0.0502 (14)	0.0635 (16)	0.0066 (11)	0.0203 (13)	0.0074 (12)
C18	0.0599 (15)	0.0530 (14)	0.0733 (17)	0.0078 (12)	0.0173 (13)	0.0046 (12)
C19	0.0606 (14)	0.0459 (14)	0.0729 (17)	0.0028 (11)	0.0161 (13)	0.0003 (12)
C20	0.0549 (14)	0.0544 (14)	0.0822 (18)	0.0061 (12)	0.0140 (13)	−0.0015 (13)
C21	0.0476 (13)	0.0589 (15)	0.0730 (17)	−0.0009 (11)	0.0119 (12)	0.0016 (12)
C22	0.0559 (14)	0.0609 (15)	0.0743 (18)	0.0046 (12)	0.0240 (13)	−0.0004 (13)
C23	0.0478 (15)	0.0671 (17)	0.106 (2)	−0.0039 (14)	0.0263 (15)	−0.0060 (15)
C24	0.0560 (17)	0.0624 (18)	0.091 (2)	−0.0045 (13)	0.0150 (15)	0.0085 (15)
N1	0.0416 (12)	0.0656 (14)	0.0920 (16)	−0.0026 (10)	0.0160 (11)	0.0063 (12)
N2	0.0434 (11)	0.0559 (12)	0.0693 (14)	0.0004 (10)	0.0183 (10)	0.0048 (10)
N3	0.0551 (13)	0.0640 (14)	0.1077 (18)	−0.0014 (11)	0.0248 (12)	−0.0043 (12)
N4	0.0485 (11)	0.0497 (12)	0.0717 (14)	0.0026 (10)	0.0239 (11)	−0.0004 (10)
N5	0.0495 (12)	0.0647 (13)	0.0877 (16)	−0.0023 (10)	0.0268 (11)	−0.0024 (11)
N6	0.0509 (11)	0.0511 (12)	0.0702 (14)	0.0031 (10)	0.0169 (10)	−0.0018 (10)
O1	0.0527 (10)	0.0750 (11)	0.1005 (14)	−0.0047 (9)	0.0280 (10)	−0.0074 (9)
O2	0.0658 (11)	0.0609 (11)	0.1015 (14)	−0.0001 (8)	0.0298 (10)	−0.0152 (9)
O3	0.0644 (10)	0.0710 (11)	0.1045 (13)	0.0199 (8)	0.0378 (10)	−0.0021 (9)
O4	0.0471 (9)	0.0732 (11)	0.1037 (13)	0.0061 (9)	0.0265 (10)	−0.0024 (10)
O5	0.0498 (9)	0.0534 (10)	0.0814 (12)	0.0018 (8)	0.0216 (8)	−0.0077 (8)
O6	0.0500 (9)	0.0625 (10)	0.1115 (14)	0.0059 (8)	0.0364 (10)	−0.0054 (8)
O1W	0.0561 (11)	0.0839 (12)	0.1221 (16)	−0.0023 (10)	0.0295 (11)	−0.0366 (10)
O2W	0.0487 (11)	0.0888 (16)	0.228 (3)	0.0104 (9)	0.0399 (14)	0.0417 (14)

### Geometric parameters (Å, °)

**Table:**

C1—C6	1.385 (2)	C16—H16A	0.9700
C1—C2	1.392 (2)	C16—H16B	0.9700
C1—C10	1.487 (2)	C17—C18	1.526 (2)
C2—C3	1.387 (2)	C17—H17A	0.9700
C2—H2	0.9300	C17—H17B	0.9700
C3—C4	1.386 (2)	C18—C19	1.528 (2)
C3—C8	1.489 (2)	C18—H18A	0.9700
C4—C5	1.377 (2)	C18—H18B	0.9700
C4—H4	0.9300	C19—C20	1.525 (2)
C5—C6	1.395 (2)	C19—H19A	0.9700
C5—C7	1.486 (2)	C19—H19B	0.9700
C6—H6	0.9300	C20—C21	1.527 (2)
C7—O1	1.205 (2)	C20—H20A	0.9700
C7—O2	1.322 (2)	C20—H20B	0.9700
C8—O3	1.214 (2)	C21—C22	1.513 (2)
C8—O4	1.307 (2)	C21—H21A	0.9700
C10—O6	1.212 (2)	C21—H21B	0.9700
C10—O5	1.311 (2)	C22—N2	1.461 (2)
C11—N5	1.316 (2)	C22—H22A	0.9700
C11—N6	1.346 (2)	C22—H22B	0.9700
C11—H11	0.9300	C23—N2	1.314 (2)
C12—N6	1.314 (2)	C23—N3	1.329 (2)
C12—N4	1.328 (2)	C23—H23	0.9300
C12—H12	0.9300	C24—N1	1.314 (2)
C13—N4	1.458 (2)	C24—N3	1.339 (2)
C13—C14	1.497 (2)	C24—H24	0.9300
C13—H13A	0.9700	N1—N2	1.3639 (19)
C13—H13B	0.9700	N4—N5	1.3605 (19)
C14—C15	1.522 (2)	O2—H2WA	0.89 (2)
C14—H14A	0.9700	O4—H4WA	0.88 (2)
C14—H14B	0.9700	O5—H5WA	0.99 (2)
C15—C16	1.519 (2)	O1W—H1AW	0.87 (2)
C15—H15A	0.9700	O1W—H1BW	0.78 (2)
C15—H15B	0.9700	O2W—H2BW	0.90 (2)
C16—C17	1.526 (2)	O2W—H2AW	0.799 (19)
			
C6—C1—C2	119.46 (16)	C18—C17—C16	113.00 (14)
C6—C1—C10	119.30 (18)	C18—C17—H17A	109.0
C2—C1—C10	121.24 (19)	C16—C17—H17A	109.0
C3—C2—C1	120.31 (18)	C18—C17—H17B	109.0
C3—C2—H2	119.8	C16—C17—H17B	109.0
C1—C2—H2	119.8	H17A—C17—H17B	107.8
C4—C3—C2	119.53 (18)	C17—C18—C19	112.76 (15)
C4—C3—C8	118.28 (18)	C17—C18—H18A	109.0
C2—C3—C8	122.2 (2)	C19—C18—H18A	109.0
C5—C4—C3	120.81 (17)	C17—C18—H18B	109.0
C5—C4—H4	119.6	C19—C18—H18B	109.0
C3—C4—H4	119.6	H18A—C18—H18B	107.8
C4—C5—C6	119.48 (18)	C20—C19—C18	112.65 (15)
C4—C5—C7	122.12 (18)	C20—C19—H19A	109.1
C6—C5—C7	118.39 (18)	C18—C19—H19A	109.1
C1—C6—C5	120.40 (17)	C20—C19—H19B	109.1
C1—C6—H6	119.8	C18—C19—H19B	109.1
C5—C6—H6	119.8	H19A—C19—H19B	107.8
O1—C7—O2	123.0 (2)	C19—C20—C21	112.14 (15)
O1—C7—C5	123.4 (2)	C19—C20—H20A	109.2
O2—C7—C5	113.60 (19)	C21—C20—H20A	109.2
O3—C8—O4	123.8 (2)	C19—C20—H20B	109.2
O3—C8—C3	122.1 (2)	C21—C20—H20B	109.2
O4—C8—C3	114.10 (18)	H20A—C20—H20B	107.9
O6—C10—O5	123.06 (17)	C22—C21—C20	110.80 (15)
O6—C10—C1	122.80 (19)	C22—C21—H21A	109.5
O5—C10—C1	114.14 (18)	C20—C21—H21A	109.5
N5—C11—N6	114.77 (18)	C22—C21—H21B	109.5
N5—C11—H11	122.6	C20—C21—H21B	109.5
N6—C11—H11	122.6	H21A—C21—H21B	108.1
N6—C12—N4	110.51 (17)	N2—C22—C21	112.51 (15)
N6—C12—H12	124.7	N2—C22—H22A	109.1
N4—C12—H12	124.7	C21—C22—H22A	109.1
N4—C13—C14	112.99 (16)	N2—C22—H22B	109.1
N4—C13—H13A	109.0	C21—C22—H22B	109.1
C14—C13—H13A	109.0	H22A—C22—H22B	107.8
N4—C13—H13B	109.0	N2—C23—N3	111.44 (18)
C14—C13—H13B	109.0	N2—C23—H23	124.3
H13A—C13—H13B	107.8	N3—C23—H23	124.3
C13—C14—C15	110.88 (15)	N1—C24—N3	116.33 (19)
C13—C14—H14A	109.5	N1—C24—H24	121.8
C15—C14—H14A	109.5	N3—C24—H24	121.8
C13—C14—H14B	109.5	C24—N1—N2	101.48 (16)
C15—C14—H14B	109.5	C23—N2—N1	109.43 (17)
H14A—C14—H14B	108.1	C23—N2—C22	130.70 (18)
C16—C15—C14	113.00 (15)	N1—N2—C22	119.71 (17)
C16—C15—H15A	109.0	C23—N3—C24	101.32 (16)
C14—C15—H15A	109.0	C12—N4—N5	109.42 (16)
C16—C15—H15B	109.0	C12—N4—C13	131.08 (18)
C14—C15—H15B	109.0	N5—N4—C13	119.50 (16)
H15A—C15—H15B	107.8	C11—N5—N4	102.27 (16)
C15—C16—C17	112.87 (15)	C12—N6—C11	103.03 (15)
C15—C16—H16A	109.0	C7—O2—H2WA	113.3 (18)
C17—C16—H16A	109.0	C8—O4—H4WA	110.7 (18)
C15—C16—H16B	109.0	C10—O5—H5WA	109.7 (17)
C17—C16—H16B	109.0	H1AW—O1W—H1BW	107 (2)
H16A—C16—H16B	107.8	H2BW—O2W—H2AW	119 (3)
			
C6—C1—C2—C3	1.0 (3)	C14—C15—C16—C17	−178.69 (16)
C10—C1—C2—C3	−178.88 (19)	C15—C16—C17—C18	178.91 (18)
C1—C2—C3—C4	−0.7 (3)	C16—C17—C18—C19	−179.83 (16)
C1—C2—C3—C8	−178.80 (19)	C17—C18—C19—C20	173.05 (17)
C2—C3—C4—C5	0.1 (3)	C18—C19—C20—C21	−175.94 (18)
C8—C3—C4—C5	178.27 (19)	C19—C20—C21—C22	176.65 (18)
C3—C4—C5—C6	0.2 (3)	C20—C21—C22—N2	176.96 (17)
C3—C4—C5—C7	−179.40 (19)	N3—C24—N1—N2	0.4 (3)
C2—C1—C6—C5	−0.6 (3)	N3—C23—N2—N1	0.5 (3)
C10—C1—C6—C5	179.24 (17)	N3—C23—N2—C22	175.8 (2)
C4—C5—C6—C1	0.0 (3)	C24—N1—N2—C23	−0.5 (2)
C7—C5—C6—C1	179.65 (18)	C24—N1—N2—C22	−176.39 (18)
C4—C5—C7—O1	177.7 (2)	C21—C22—N2—C23	16.7 (3)
C6—C5—C7—O1	−1.9 (3)	C21—C22—N2—N1	−168.43 (18)
C4—C5—C7—O2	−2.3 (3)	N2—C23—N3—C24	−0.2 (3)
C6—C5—C7—O2	178.06 (18)	N1—C24—N3—C23	−0.1 (3)
C4—C3—C8—O3	3.1 (3)	N6—C12—N4—N5	−0.1 (3)
C2—C3—C8—O3	−178.8 (2)	N6—C12—N4—C13	−179.88 (19)
C4—C3—C8—O4	−176.32 (19)	C14—C13—N4—C12	0.9 (3)
C2—C3—C8—O4	1.8 (3)	C14—C13—N4—N5	−178.88 (18)
C6—C1—C10—O6	4.9 (3)	N6—C11—N5—N4	0.3 (3)
C2—C1—C10—O6	−175.3 (2)	C12—N4—N5—C11	−0.1 (2)
C6—C1—C10—O5	−174.98 (18)	C13—N4—N5—C11	179.71 (18)
C2—C1—C10—O5	4.9 (3)	N4—C12—N6—C11	0.3 (2)
N4—C13—C14—C15	−177.54 (17)	N5—C11—N6—C12	−0.3 (3)
C13—C14—C15—C16	−178.73 (18)		

### Hydrogen-bond geometry (Å, °)

**Table:**

*D*—H···*A*	*D*—H	H···*A*	*D*···*A*	*D*—H···*A*
O2—H2WA···O1W	0.89 (2)	1.66 (2)	2.549 (2)	177 (3)
O4—H4WA···O2W	0.88 (2)	1.70 (2)	2.571 (2)	169 (3)
O5—H5WA···N6^i^	0.99 (2)	1.59 (2)	2.5750 (19)	174 (3)
O1W—H1AW···O3^ii^	0.87 (2)	1.86 (2)	2.711 (2)	163 (3)
O1W—H1BW···O1^iii^	0.78 (2)	1.99 (2)	2.757 (2)	170 (3)
O2W—H2BW···N3^ii^	0.90 (2)	2.12 (2)	2.875 (2)	140 (3)
O2W—H2AW···O6^iv^	0.80 (2)	2.05 (2)	2.841 (2)	170 (3)

Symmetry codes: (i) −*x*+1, −*y*+3, −*z*+2; (ii) −*x*+1, −*y*+1, −*z*+1; (iii) −*x*, −*y*+1, −*z*+1; (iv) *x*+1, *y*, *z*.
